# Additive effectiveness of acrylonitrile‐co‐methallyl sulfonate surface‐treated membranes in the treatment of pneumonia: A propensity score‐matched retrospective cohort study

**DOI:** 10.1111/aor.14435

**Published:** 2022-11-08

**Authors:** Kentaro Hayashi, Yusuke Sasabuchi, Hiroki Matsui, Mikio Nakajima, Hiroyuki Ohbe, Kiyohide Fushimi, Kazuyuki Ono, Hideo Yasunaga

**Affiliations:** ^1^ Department of Emergency and Critical Care Medicine Dokkyo Medical University Tochigi Japan; ^2^ Data Science Center Jichi Medical University Tochigi Japan; ^3^ Department of Clinical Epidemiology and Health Economics, School of Public Health The University of Tokyo Tokyo Japan; ^4^ Emergency and Critical Care Center Tokyo Metropolitan Hiroo Hospital Tokyo Japan; ^5^ Department of Health Policy and Informatics Tokyo Medical and Dental University Graduate School of Medicine Tokyo Japan

**Keywords:** acrylonitrile‐co‐methallyl sulfonate membrane, continuous renal replacement therapy, cytokine adsorption therapy, pneumonia, sepsis

## Abstract

**Background:**

The acrylonitrile‐co‐methallyl sulfonate surface‐treated (AN69ST) membrane has cytokine adsorption capacity and is used for treating sepsis. This study aimed to compare the effects of continuous renal replacement therapy (CRRT) using the AN69ST membrane with those of CRRT using other membranes for patients with pneumonia‐associated sepsis.

**Methods:**

This retrospective, propensity score‐matched, cohort study was based on a nationwide Japanese inpatient database. We included data from adults hospitalized with a primary diagnosis of pneumonia, who received CRRT using either the AN69ST membrane or another membrane within 2 days of admission, and who were discharged from the hospitals between September 2014, and March 2017. Propensity score matching was used to compare in‐hospital mortality between the two groups.

**Results:**

Eligible patients (*N* = 2393) were categorized into an AN69ST group (*N* = 631) and a non‐AN69ST group (*N* = 1762). The overall in‐hospital mortality rate was 38.9%. Among the 545 propensity‐matched patient pairs, the in‐hospital mortality rate was significantly lower in the AN69ST group than in the non‐AN69ST group (35.8 vs. 41.8%, *p* = 0.046).

**Conclusions:**

Among patients with pneumonia‐associated sepsis treated with CRRT, CRRT with the AN69ST membrane was associated with a significantly lower in‐hospital mortality than CRRT with standard membranes.

## Graphical abstract

Continuous renal replacement therapy (CRRT) was studied in patients with pneumonia. CRRT using the AN69ST membrane was compared to conventional CRRT. The in‐hospital mortality rate was significantly lower in the AN69ST group. log rank *p* = 0.016.

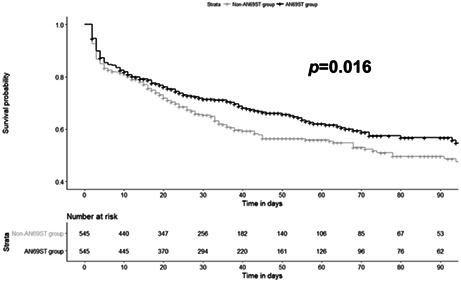

## 1 INTRODUCTION

Sepsis causes dysfunction of various organs and can lead to death in critically ill patients.^1^ Pneumonia is the most common cause of sepsis and death worldwide.^2^ Cytokines may play an essential role in the mechanism of organ dysfunction and mortality associated with sepsis.^3^, ^4^ The extent of systemic cytokine elevation has been suggested to reflect the disease severity of patients with pneumonia.^5^ Although continuous renal replacement therapy (CRRT) removes cytokines and other inflammatory mediators,^6^, ^7^ it does not improve clinical outcomes, regardless of the applied/high‐volume dose.^8^, ^9^ In recent years, various new approaches based on CRRT, such as endotoxin adsorption therapy using polymyxin B hemoperfusion^10^ and cytokine removal therapy using standard CRRT membranes,^11^, ^12^, ^13^ have been introduced to improve the prognosis of sepsis with hypercytokinemia.^14^, ^15^ However, these approaches, such as high‐volume continuous hemofiltration or cytokine and/or endotoxin removal with polymyxin B hemoperfusion, have not been shown to improve the prognosis of sepsis to date.^8^, ^13^, ^14^, ^15^ A meta‐analysis suggests that plasma exchange and hemoadsorption are potentially effective blood purification methods for the treatment of sepsis.^16^ There is a possibility that CRRT with blood adsorption therapy may be effective in the treatment of COVID‐19.^17^

Acrylonitrile‐co‐methallyl sulfonate surface‐treated (AN69ST) membrane (sepXiris™, Baxter), one of the membranes used for CRRT, has been used for cytokine adsorption therapy in Japan since September 2014. The AN69ST membrane has a hydrogel structure, enabling cytokine adsorption, not only on the membrane surface but also within the bulk layer, thereby exhibiting an increased capacity for cytokine removal in vitro.^18^, ^19^ Therefore, the standard CRRT membrane has been widely replaced by the AN69ST membrane to absorb cytokines in critically ill patients in Japan, regardless of the cause of sepsis. However, only a few clinical studies^20^, ^21^, ^22^, ^23^, ^24^ on the AN69ST membrane have been reported, and the clinical effectiveness of the AN69ST membrane remains unclear.

## 2 METHODS

### 2.1 Study aim, design, and data source

This retrospective study aimed to investigate the clinical effects of the AN69ST membrane, compared with those of standard CRRT membranes in patients with pneumonia‐associated sepsis, using data from the Japanese Diagnostic Procedure Combination Database.^25^ This database contains administrative claims data and clinical information. All 82 academic hospitals in Japan are required to provide information to this database. However, participation by community hospitals is voluntary. The database includes the following information: age, sex, and diagnosis (primary diagnosis at admission, comorbidities at diagnosis, and postadmission complications) recorded by the International Classification of Diseases, 10th Revision, (ICD‐10) codes.^26^ Text data in Japanese, such as transfer transportation mode (e.g., ambulance), medical procedures (including types of surgery and the dates on which they were conducted, daily records of drug administration, and devices used), date of admission and discharge, and discharge status, were included. The database was structured explicitly to differentiate between preadmission comorbidities and postadmission complications. All clinical data for each patient were recorded at discharge by attending physicians (see Table S1). This study was approved by the Institutional Review Board of the University of Tokyo. The need for informed consent was waived because this was a retrospective study, using anonymized data.

### 2.2 Patient selection

We identified patients in the database with pneumonia as the primary diagnosis on admission and with a hospital discharge date between September 1, 2014, and March 31, 2017. We then included patients with pneumonia‐associated sepsis according to the following criteria: (1) a primary diagnosis of pneumonia on admission (ICD‐10 codes J13.x–J18.x)^27^, ^28^ and (2) patients who had undergone CRRT with the AN69ST membrane or a standard membrane within 2 days of admission. The exclusion criteria were as follows: (1) age < 18 years, (2) death within 2 days of admission, and (3) administration of CRRT with the AN69ST membrane and a standard membrane after 2 days of admission.

### 2.3 Exposure and outcome

The exposure of interest was AN69ST‐CRRT (AN69ST group) and standard CRRT (non‐AN69ST group) within 2 days of admission. The primary outcome was in‐hospital mortality. The 30‐day mortality rate, length of stay, total cost (Great Britain pound, GBP) during admission, and CRRT period (the duration for which CRRT was performed) were secondary outcomes.

### 2.4 Other variables

Other variables included age, sex, body mass index, fiscal year, transfer by ambulance, hospital type, hospital size, unit type, comorbidities, blood transfusion, requirement for mechanical ventilation, use of cardiovascular agents, use of drugs for disseminated intravascular coagulation, use of immunoglobulin or steroids, use of polymyxin B‐immobilized fiber column‐direct hemoperfusion, hemodialysis, and complications such as organ failure on admission (based on renal, cardiovascular, neurological, hematological, and hepatic status). Hospital volume was defined as the average annual number of patients with pneumonia who had undergone CRRT with any type of membrane within 2 days of admission. Comorbidities on admission were extracted for each component of the Charlson Comorbidity Index, using algorithms developed by Quan et al.^29^ Data were extracted from the ICD‐10 codes of complications and the procedures listed in the supplementary table. Body mass index values were categorized as missing when weight and height values were unavailable.

### 2.5 Statistical analyses

We used propensity score methods, which have been used in several previous retrospective observational studies to compare groups with similar characteristics without specification of the relationship between confounders and outcomes.^30^ Similarly, we used propensity score matching^31^ to adjust for differences in baseline characteristics and the severity of the condition on admission between the AN69ST and non‐AN69ST groups. To estimate the probability of receiving AN69ST‐CRRT or another standard CRRT, a propensity score was calculated for each patient using multivariable logistic regression analysis. Each patient in the AN69ST group was individually matched with a patient in the non‐AN69ST group, based on nearest‐neighbor matching, without replacement. The caliper was set at 0.2 for the SD of the propensity scores. The balance between the two groups was compared using the standardized mean difference (SMD), and SMD <0.1 was considered a negligible imbalance. The outcomes between the two groups were compared using Fisher's exact test for in‐hospital mortality and the Mann–Whitney *U* test for the length of stay and total cost (GBP, calculated 1GBP = 165JPY). Kaplan–Meier survival curves were plotted for the AN69ST group and the non‐AN69ST group, and the log‐rank test was used to compare the survival curves.

We conducted subgroup analyses on all baseline characteristics and in‐hospital mortality using the Breslow‐Day test for categorical variables and generalized linear models for continuous variables. *p* values of <0.05 were considered statistically significant. All analyses were conducted using SPSS version 22 (IBM Corp, Armonk, NY, US) and R version 3.1.3 (The R Foundation for Statistical Computing, Vienna, Austria).

## 3 RESULTS

### 3.1 Patients

**FIGURE 1:**
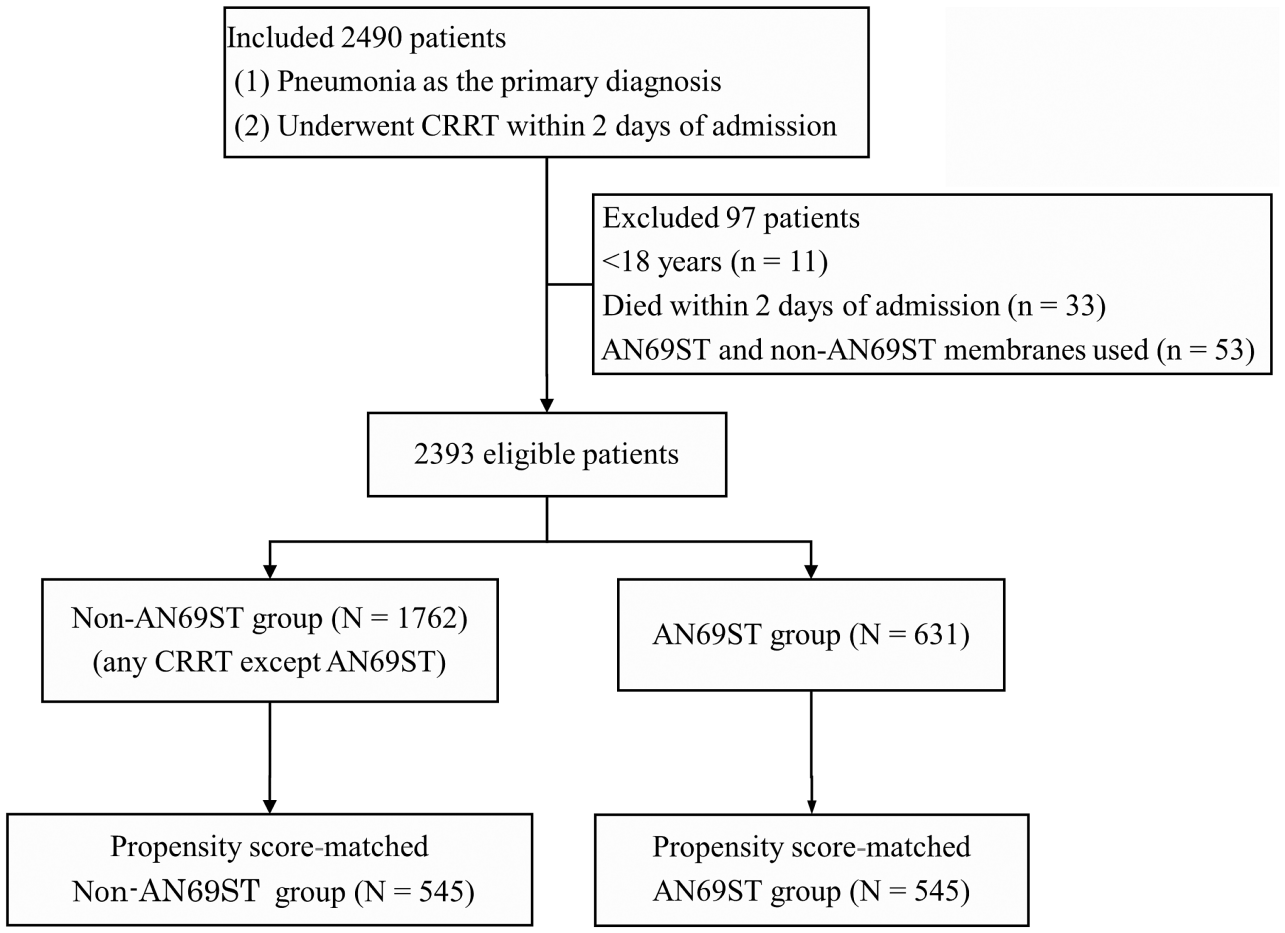
CONSORT flow chart. AN69ST, acrylonitrile‐co‐methallyl sulfonate surface‐treated membrane; CRRT, continuous renal replacement therapy.

A total of 2393 patients were included in the study (Figure 1), 631 of whom were assigned to the AN69ST group and 1762 to the non‐AN69ST group. The characteristics of the patients before and after propensity score matching are presented in Table 1. After propensity score matching, the baseline patient characteristics were well balanced between the two groups.

**TABLE 1:** Baseline patient characteristics before and after propensity score matching

Variable	Pre‐matching cohort			Propensity score‐matched cohort		
	Non‐AN69ST group	AN69ST group	SMD	Non‐AN69ST group	AN69ST group	SMD
	(*N* = 1762)	(*N* = 631)		(*N* = 545)	(*N* = 545)	
Fiscal year, *n* (%)			0.57			0.03
2014	501 (28.4)	64 (10.1)		70 (12.8)	62 (11.4)	
2015	684 (38.8)	217 (34.4)		177 (32.5)	203 (37.2)	
2016	577 (32.7)	350 (55.5)		298 (54.7)	280 (51.4)	
Age (years), mean (SD)	72.61 (12.4)	73.27 (12.5)	0.05	72.75 (12.1)	73.30 (12.6)	0.04
Sex (female), *n* (%)	654 (37.1)	228 (36.1)	0.02	198 (36.3)	201 (36.9)	0.01
BMI (kg/m^2^)			0.11			0.04
<18.5	348 (19.8)	122 (19.3)		99 (18.2)	102 (18.7)	
18.5–22.5	651 (36.9)	230 (36.5)		208 (38.2)	203 (37.2)	
22.5–25	301 (17.1)	106 (16.8)		96 (17.6)	94 (17.2)	
25–30	235 (13.3)	105 (16.6)		84 (15.4)	86 (15.8)	
≥30	67 (3.8)	18 (2.9)		16 (2.9)	14 (2.6)	
Missing	160 (9.1)	50 (7.9)		42 (7.7)	46 (8.4)	
Transferred by ambulance, *n* (%)	1319 (74.9)	490 (78.0)	0.07	428 (78.5)	413 (75.8)	0.07
Hospital type (academic), *n* (%)	544 (30.9)	179 (28.4)	0.06	168 (30.8)	158 (29.0)	0.04
Hospital volume, mean (SD)	4.25 (3.5)	7.07 (6.7)	0.53	5.40 (4.7)	5.34 (5.0)	0.01
Type of unit						
ICU, *n* (%)	846 (48.0)	311 (49.3)	0.03	260 (47.7)	251 (46.1)	0.03
HCU, *n* (%)	119 (6.8)	35 (5.5)	0.05	34 (6.2)	34 (6.2)	<0.01
Comorbidity, *n* (%)						
Liver disease	90 (5.1)	32 (5.1)	<0.01	29 (5.3)	25 (4.6)	0.03
Renal disease	492 (27.9)	123 (19.5)	0.2	122 (22.4)	117 (21.5)	0.02
Myocardial infarction	34 (1.9)	5 (0.8)	0.1	4 (0.7)	4 (0.7)	<0.01
Congestive heart failure	225 (12.8)	75 (11.9)	0.03	70 (12.8)	61 (11.2)	0.05
Peripheral vascular disease	30 (1.7)	13 (2.1)	0.03	7 (1.3)	8 (1.5)	0.02
Cerebrovascular disease	66 (3.7)	28 (4.4)	0.04	20 (3.7)	20 (3.7)	<0.01
Hemiplegia/paraplegia	2 (0.1)	0 (0.0)	0.05	0 (0.0)	0 (0.0)	<0.01
Dementia	39 (2.2)	19 (3.0)	0.05	16 (2.9)	17 (3.1)	0.01
Chronic pulmonary disease	44 (2.5)	20 (3.2)	0.04	16 (2.9)	18 (3.3)	0.02
Rheumatic disease	39 (2.2)	14 (2.2)	<0.01	13 (2.4)	13 (2.4)	<0.01
Peptic ulcer	153 (8.7)	57 (9.0)	0.01	45 (8.3)	40 (7.3)	0.03
DM without complication	162 (9.2)	62 (9.8)	0.02	54 (9.9)	50 (9.2)	0.03
DM with complication	90 (5.1)	23 (3.6)	0.07	21 (3.9)	20 (3.7)	0.01
AIDS	1 (0.1)	0 (0.0)	0.03	0 (0.0)	0 (0.0)	<0.01
Malignancy	199 (11.3)	78 (12.4)	0.03	56 (10.3)	65 (11.9)	0.05
Metastatic cancer	23 (1.3)	12 (1.9)	0.05	13 (2.4)	9 (1.7)	0.05
Blood transfusion, *n* (%)						
Red blood cells	722 (41.0)	302 (47.9)	0.14	254 (46.6)	244 (44.8)	0.04
Fresh frozen plasma	646 (36.7)	279 (44.2)	0.15	232 (42.6)	236 (43.3)	0.02
Platelet	221 (12.5)	71 (11.3)	0.04	60 (11.0)	59 (10.8)	<0.01
Cardiovascular agents, *n* (%)						
Dopamine	582 (33.0)	174 (27.6)	0.12	143 (26.2)	154 (28.3)	0.05
Dobutamine	267 (15.2)	114 (18.1)	0.08	112 (20.6)	104 (19.1)	0.04
Noradrenaline	1314 (74.6)	551 (87.3)	0.33	463 (85.0)	472 (86.6)	0.05
Adrenaline	204 (11.6)	100 (15.8)	0.12	73 (13.4)	83 (15.2)	0.05
Vasopressin	329 (18.7)	164 (26.0)	0.18	130 (23.9)	120 (22.0)	0.04
Milrinone	21 (1.2)	7 (1.1)	<0.01	5 (0.9)	6 (1.1)	0.02
Oral catecholamine	23 (1.3)	4 (0.6)	0.07	3 (0.6)	4 (0.7)	0.02
Intervention, *n* (%)						
Coadministered DIC drug	1614 (91.6)	591 (93.7)	0.08	504 (92.5)	510 (93.6)	0.04
Immunoglobulin	689 (39.1)	249 (39.5)	<0.01	229 (42.0)	231 (42.4)	<0.01
Oral steroids	25 (1.4)	5 (0.8)	0.06	3 (0.6)	4 (0.7)	0.02
Intravenous steroids	701 (39.8)	271 (42.9)	0.06	242 (44.4)	243 (44.6)	<0.01
Mechanical ventilation	1360 (77.2)	514 (81.5)	0.11	458 (84.0)	444 (81.5)	0.07
PMX‐DHP	766 (43.5)	274 (43.4)	<0.01	243 (44.6)	247 (45.3)	0.02
Hemodialysis	53 (3.0)	14 (2.2)	0.05	14 (2.6)	13 (2.4)	0.01
Complications, *n* (%)						
AKI after admission	389 (22.1)	177 (28.1)	0.14	138 (25.3)	143 (26.2)	0.02
Cardiovascular on admission	165 (9.4)	74 (11.7)	0.08	66 (12.1)	65 (11.9)	<0.01
Neurological status on admission	12 (0.7)	2 (0.3)	0.05	0 (0.0)	1 (0.2)	0.06
Hematological status on admission	349 (19.8)	139 (22.0)	0.06	122 (22.4)	123 (22.6)	<0.01
Hepatic status on admission	14 (0.8)	5 (0.8)	<0.01	2 (0.4)	4 (0.7)	0.05
Renal status on admission	436 (24.7)	173 (27.4)	0.06	141 (25.9)	139 (25.5)	<0.01

*Note*: Data are presented as numbers (%) unless stated otherwise.

Abbreviations: AIDS, acquired immunodeficiency syndrome; AKI, acute kidney injury; AN69ST, AN69 surface treatment; BMI, body mass index; DIC, disseminated intravascular coagulation; DM, diabetes mellitus; HCU, high care unit; ICU, intensive care unit; PMX‐DHP, polymyxin B‐immobilized fiber column‐direct hemoperfusion; SMD, standardized mean difference.

### 3.2 Outcomes

**FIGURE 2:**
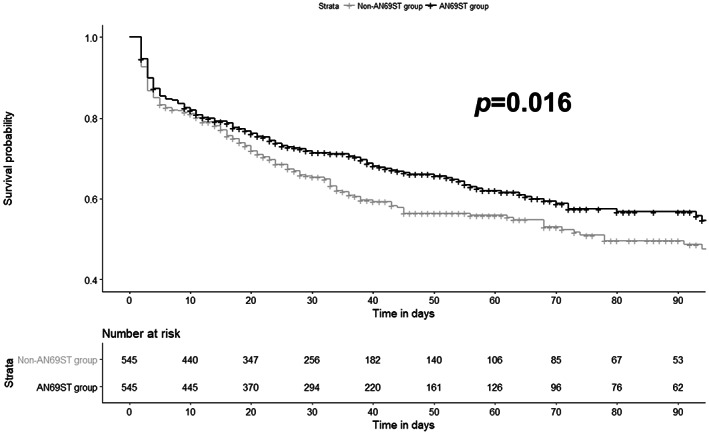
Kaplan–Meier survival curves for 90‐day mortality according to the group after propensity score matching. AN69ST, acrylonitrile‐co‐methallyl sulfonate surface‐treated membrane.

The overall in‐hospital mortality in this study was 38.9% (930/2393). The outcomes before and after propensity score matching are presented in Table 2. There was a significant difference in in‐hospital mortality between the AN69ST group and the non‐AN69ST group after propensity score matching (35.8% vs. 41.8%; *p* = 0.046). The Kaplan–Meier survival curve for the 90‐day mortality rate of the two groups after propensity score matching is presented in Figure 2. As evident from the data, the 30‐day mortality was significantly different between the AN69ST group and the non‐AN69ST group (log‐rank test, *p* = 0.02). There was also a significant difference in the length of stay between the two groups after propensity score matching (32 and 27 days for the AN69ST group and the non‐AN69ST group, respectively; *p* = 0.03). There was a significant difference in the total cost between the two groups after propensity score matching (23 510.8 and 21 116.7 GBP for the AN69ST group and the non‐AN69ST group, respectively; *p* = 0.02). There was no significant difference in the CRRT period between the two groups (3 and 3 days for the AN69ST group and non‐AN69ST group, respectively; *p* = 0.573).

**TABLE 2:** Outcomes before and after propensity score matching

	Prematching cohort			Propensity score‐matched cohort		
	Non‐AN69ST group (*n* = 1762)	AN69ST group (*n* = 631)	*p*‐value	Non‐AN69ST group (*n* = 545)	AN69ST group (*n* = 545)	*p*‐value
In‐hospital mortality, *n* (%)	700 (39.7)	230 (36.5)	0.16	228 (41.8)	195 (35.8)	0.046
Length of stay (days), median (IQR)	29 (14–53)	32 (16–56)	0.12	27 (14–56)	32 (15–56)	0.03
Total cost (GBP), median (IQR)	20 676.4 (13 015.7–31 794.7)	23 488.2 (14 187.6–35 194.8)	<0.01	21 116.7 (12 743.8–31 762.4)	23 510.8 (14 064.7–34 782.4)	0.02
CRRT period (days), median (IQR)	3 (2–7)	3 (2–6)	0.31	3 (2–6)	3 (2–6)	0.58

Abbreviations: AN69ST, acrylonitrile‐co‐methallyl sulfonate surface‐treated membrane; CRRT, continuous renal replacement therapy; GBP, Great Britain pound (calculated 1GBP = 165JPY); IQR, interquartile range.

### 3.3 Subgroup analysis

The interaction between representative variables associated with in‐hospital mortality and the outcomes is presented in Table 3. No significant interactions were observed.

**TABLE 3:** Results of subgroup analyses for in‐hospital mortality and length of stay

Variables	*p*‐value for interaction	
	In‐hospital mortality	Length of stay
BMI	0.34	0.07
Admission by ambulance	0.53	0.30
Renal complication at admission	0.07	0.68
PMX‐DHP	0.10	0.58
IRRT	0.93	0.15
Cardiovascular agents within 2 days	0.18	0.24
Mechanical ventilation within 2 days	0.36	0.30
Hospitalization to the intensive care unit	0.10	0.41
Academic hospital	0.73	0.71
Malignancy as a comorbidity	0.70	0.13

Abbreviations: BMI, body mass index; IRRT, intermittent renal replacement therapy; PMX‐DHP, polymyxin B‐immobilized fiber column‐direct hemoperfusion.

## 4 DISCUSSION

This study investigated the additive effect of the AN69ST membrane in the treatment of pneumonia‐associated sepsis. Compared with CRRT with standard membranes, CRRT with the AN69ST membrane appeared to reduce mortality in patients with pneumonia‐associated sepsis. Several aspects of this study differed from those of previous studies that investigated the effect of the AN69ST membrane compared with that of the standard CRRT membrane.^21^, ^22^, ^23^, ^24^

First, our previous study, which investigated the additive effect of the AN69ST membrane in patients with panperitonitis due to lower gastrointestinal perforation, did not show a significant difference in outcomes between the AN69ST membrane and the standard CRRT membrane groups.^24^ The characteristics of patients included in this study also differed from those of patients in the previous study. Most pathogenic microorganisms responsible for panperitonitis are gram‐negative bacilli.^32^ Conversely, pneumonia‐causing pathogenic microorganisms, particularly those that cause community‐acquired pneumonia, are nonbacterial or gram‐positive cocci^33^ that do not produce endotoxins.^34^ Infections caused by gram‐negative bacilli have a larger quantity of endotoxin production than those caused by nonbacterial or gram‐positive cocci^35^; additionally, endotoxins induce cytokine production.^36^ As the AN69ST membrane has been reported to adsorb cytokines,^21^, ^22^, ^23^, ^24^ it is possible that its effect on mortality reduction in pneumonia, as observed in this study, can be attributed particularly to infections with gram‐positive cocci unlike those seen in lower gastrointestinal perforations. Future research on the effectiveness of the AN69ST membrane with further consideration for the disease type or microorganism identification is warranted.

Second, the timing of cytokine removal therapy may also have contributed to the difference between the results of previous studies and those of this study. Several articles have reported that initiating cytokine adsorption therapy within 24 h after diagnosis may improve patient prognosis.^37^ Reports on the results of several previous studies^20^, ^21^, ^22^, ^23^ that investigated the effect of the AN69ST membrane did not explicitly report the timing of CRRT initiation. In this study, we only included patients in whom CRRT was initiated within 2 days of admission.

Third, the severity of sepsis among patients in this study may have been lower than that in previous studies. In this study, the overall in‐hospital mortality rate was 38.9%, and the 30‐day mortality rate was approximately 30%. In contrast, the overall mortality rate was 51.4% in one study,^21^ and the 28‐day mortality rate was 45.9% in another study.^24^ It is difficult to directly compare the present study with previous studies because the treatment strategy for sepsis varies by the source of infection, however, based on these results, the AN69ST membrane may only be effective in patients with mild to moderate grades of sepsis.

This study has several limitations. First, this is a retrospective study using the Japanese Diagnostic Procedure Combination Database. As it is a clinical administrative claims database, it has no data on laboratory tests, vital signs, culture results, and severity scores such as the Acute Physiology and Chronic Health Evaluation (APACHE) II score. Although we adjusted for several potential confounding factors using propensity score matching, residual confounders, including laboratory results and vital signs, might have biased the results. Second, because sepsis is one of the indications for CRRT with the AN69ST membrane, the proportion of patients with acute kidney injury may have been smaller in the AN69ST group than in the non‐AN69ST group. The aim of the CRRT was not documented in the database, and, as such, it was unclear whether the AN69ST membrane was used for the indication of blood purification or renal replacement. This may have favorably biased the results toward lower mortality rates among patients in the AN69ST membrane group. Third, we were unable to differentiate the types of membranes used in the non‐AN69ST group. Fourth, the AN69ST membrane has been reported to adsorb nafamostat mesylate,^38^ which is used as an anticoagulant. However, we did not assess the potential adverse events and complications of the AN69ST membrane in the present study. Finally, despite the use of propensity score matching, there is still a possibility of residual confounding.

## 5 CONCLUSION

In conclusion, this retrospective cohort study suggested that in patients with pneumonia‐associated sepsis, the AN69ST membrane was significantly associated with decreased in‐hospital mortality and 30‐day mortality compared to standard CRRT membranes. Further research on the effectiveness of the AN69ST membrane with consideration for the disease or microorganism is required to determine the overall clinical effectiveness of AN69ST membranes in sepsis.

## AUTHOR CONTRIBUTIONS

Kentarou Hayashi: Methodology, Investigation, Formal analysis, Writing—Original Draft; Yusuke Sasabuchi: Methodology, Investigation, Formal analysis, Writing—Original Draft; Hiroki Matsui: Methodology, Investigation, Formal analysis, Writing—Original Draft; Mikio Nakajima: Methodology, Investigation, Formal analysis, Writing—Original Draft; Hiroyuki Ohbe: Methodology, Investigation, Formal analysis, Writing—Original Draft; Kiyohide Fushimi: Methodology, Investigation, Writing—Review and Editing; Kazuyuki Ono: Supervision, Writing—Review and Editing; Hideo Yasunaga: Methodology, Investigation, Formal analysis, Writing – Original Draft, Supervision.

## CONFLICT OF INTEREST

The authors declare that they have no competing interests.

## ETHICS STATEMENT

The research was approved by the Institutional Review Board of the University of Tokyo. Patient consent was waived owing to the use of retrospective, anonymized data.

## Supporting information


Table S1
