# Supplementary material for: Additive effectiveness of acrylonitrile‐co‐methallyl sulfonate surface‐treated membranes in the treatment of pneumonia: A propensity score‐matched retrospective cohort study
Source: Artif Organs. 2022 Nov 8;47(2):408–16. doi: 10.1111/aor.14435 (PMC10099711; doi:10.1111/aor.14435)
Supplement: Supplementary file 1 — Table S1 [file AOR-47-408-s001.docx]

**Supplementaly Table**

ICD-10 codes, Japanese procedure codes, and claims data used to define organ failure

| Complications | ICD-10 codes | Statas |
| --- | --- | --- |
| Cardiovascular | R57 I95 | Coexisting disease at admission |
| Neurologic | G93.4 F05 | Coexisting disease at admission |
| Haematologic | D65 D69.5 D69.6 D69.8 D69.9 | Coexisting disease at admission |
| Hepatic | K72.0 K76.3 | Coexisting disease at admission |
| Renal (Renal complication at admission) | N17 | Coexisting disease at admission |
| AKI | N17 | Developed after admission |

AKI, acute kidney injury; ICD-10, International Classification of Diseases, Tenth Revision.
